# PLACEKEEPING AND SENSE OF PLACE: USING PHOTOVOICE TO HIGHLIGHT NEIGHBORHOOD DISPLACEMENT

**DOI:** 10.1093/geroni/igz038.096

**Published:** 2019-11-08

**Authors:** Shellae Versey

**Affiliations:** Wesleyan University, Middletown, Connecticut, United States

## Abstract

There has been growing interest in the use of community-based participatory research (CBPR) in gerontology. Photovoice, one of several qualitative methods utilized in CBPR, pairs participants with photography to identify and represent issues of importance. This paper explores photovoice as a tool for meaning making and preserving a ‘sense of place’ in a gentrifying context in New York City. Older residents describe pending neighborhood displacement due to gentrification using photographs. Using these themes and a range of visual media, older adults mobilize preservation and resistance efforts to gentrification. The paper concludes with implications and directions for future research.

